# A3 TARGETING PROLINE METABOLISM TO OVERCOME TREATMENT RESISTANCE IN ESOPHAGEAL CANCER

**DOI:** 10.1093/jcag/gwab049.002

**Published:** 2022-02-21

**Authors:** J Douchin, L Nogueira de Almeida, A Gonneaud, F Boisvert, A Dufour, V Giroux

**Affiliations:** 1 Biologie cellulaire, Université de Sherbrooke, Sherbrooke, QC, Canada; 2 University of Calgary Cumming School of Medicine, Calgary, AB, Canada; 3 Department of Anatomy and Cell Biology, Faculty of Medicine and Health Sciences, Université de Sherbrooke, Sherbrooke, QC, Canada; 4 Anatomy and Cell Biology, Université de Sherbrooke, Sherbrooke, QC, Canada; 5 Immunology and Cell Biology, Université de Sherbrooke, Sherbrooke, QC, Canada

## Abstract

**Background:**

Patients with esophageal malignancy have a 5-year survival rate of only 14% in Canada. This high mortality rate is due to three factors: late diagnosis, difficulty to surgically remove the tumor due to its localization and treatment resistance. Treatment resistance has been ascribed to the presence of cancer stem cells (CSCs) inside the tumor. However, no treatment specifically directed against CSCs is available to patients. Therefore, targeting CSCs is a promising strategy to improve survival of patients with esophageal squamous cell carcinoma (ESCC), the most common type of esophageal cancer worldwide.

**Aims:**

Herein, we developed an unbiased approach to identify new players in chemotherapy and radiotherapy resistance in ESCC.

**Methods:**

We established radioresistant (RR), chemoresistant (CR) and radiochemoresistant (RCR) human ESCC cell lines using weekly radiation and/or continuous treatment with increasing doses of chemotherapeutic agent 5-FU. We validated that the process of resistance acquisition correlates with enrichment in CSCs as revealed by higher ALDH1 expression, and increased proportion of ALDH1^high^ cells and CD24^high^/CD44^high^ cells in flow cytometry. We then used a proteomic approach to identify new players in treatment resistance.

**Results:**

Interestingly, pathway analysis demonstrated enrichment in energy metabolism as well as amino acid metabolism. Seahorse assays showed a more quiescent metabolism in all three types of resistant cells compared to the control cell line. More precisely, resistant cell lines have a lower respiration rate than control cell line, while glycolysis remains unchanged. Surprisingly, our results show a metabolic rewiring very different from the well-known Warburg effect. To further characterise these metabolic changes, we performed an unbiased metabolomic pilot study and confirmed a decrease in amino acid levels such as proline, in resistant cell lines. Preliminary data show that when cultured in DMEM with proline addition, CD44^high^/CD24^high^ cell proportion is decreased in control and RR cell lines suggesting that proline is a key regulator of CSC population in ESCC.

**Conclusions:**

To conclude, our results suggest an important role of metabolism in ESCC treatment resistance. This study is a first step towards the identification of new targets to fight treatment resistance in ESCC patients.

**Funding Agencies:**

CAG, CIHRCanada research chair TIER 2

